# Interleukin 32 expression in human melanoma

**DOI:** 10.1186/s12967-019-1862-y

**Published:** 2019-04-05

**Authors:** Helicia Paz, Jennifer Tsoi, Anusha Kalbasi, Catherine S. Grasso, William H. McBride, Dörthe Schaue, Lisa H. Butterfield, Deena M. Maurer, Antoni Ribas, Thomas G. Graeber, James S. Economou

**Affiliations:** 1 Department of Surgery, University of California, Los Angeles, 10833 Le Conte Ave, Los Angeles, CA 90095 USA; 2 Department of Microbiology, Immunology, and Molecular Genetics, University of California, Los Angeles, CA 90095 USA; 3 Department of Molecular and Medical Pharmacology, Crump Institute for Molecular Imaging, University of California, Los Angeles, CA 90095 USA; 4 Department of Radiation Oncology, David Geffen School of Medicine, University of California, Los Angeles, CA 90095 USA; 5 Department of Medicine, David Geffen School of Medicine, University of California, Los Angeles, CA 90095 USA; 6 Jonsson Comprehensive Cancer Center, University of California, Los Angeles, CA 90095 USA; 7 Department of Immunology, University of Pittsburgh, Pittsburgh, PA 15213 USA; 8 Department of Medicine, University of Pittsburgh Cancer Institute, Pittsburgh, PA 15213 USA; 9 Department of Surgery, University of Pittsburgh Cancer Institute, Pittsburgh, PA 15213 USA; 10 Department of Clinical and Translational Science, University of Pittsburgh, Pittsburgh, PA 15213 USA

**Keywords:** Interleukin 32 (IL32), Melanoma dedifferentiation, IL32 transcriptional regulation, Immune infiltration, Myeloid polarization

## Abstract

**Background:**

Various proinflammatory cytokines can be detected within the melanoma tumor microenvironment. Interleukin 32 (IL32) is produced by T cells, NK cells and monocytes/macrophages, but also by a subset of melanoma cells. We sought to better understand the biology of IL32 in human melanoma.

**Methods:**

We analyzed RNA sequencing data from 53 in-house established human melanoma cell lines and 479 melanoma tumors from The Cancer Genome Atlas dataset. We evaluated global gene expression patterns associated with IL32 expression. We also evaluated the impact of proinflammatory molecules TNFα and IFNγ on IL32 expression and dedifferentiation in melanoma cell lines in vitro. In order to study the transcriptional regulation of IL32 in these cell lines, we cloned up to 10.5 kb of the 5′ upstream region of the human IL32 gene into a luciferase reporter vector.

**Results:**

A significant proportion of established human melanoma cell lines express IL32, with its expression being highly correlated with a dedifferentiation genetic signature (high AXL/low MITF). Non IL32-expressing differentiated melanoma cell lines exposed to TNFα or IFNγ can be induced to express the three predominant isoforms (α, β, γ) of IL32. *Cis*-acting elements within this 5′ upstream region of the human IL32 gene appear to govern both induced and constitutive gene expression. In the tumor microenvironment, IL32 expression is highly correlated with genes related to T cell infiltration, and also positively correlates with high AXL/low MITF dedifferentiated gene signature.

**Conclusions:**

Expression of IL32 in human melanoma can be induced by TNFα or IFNγ and correlates with a treatment-resistant dedifferentiated genetic signature. Constitutive and induced expression are regulated, in part, by *cis*-acting sequences within the 5′ upstream region.

**Electronic supplementary material:**

The online version of this article (10.1186/s12967-019-1862-y) contains supplementary material, which is available to authorized users.

## Background

Though first described in 1992 as NK4, interleukin 32 (IL32) [1, 2] was recharacterized in 2005 as a proinflammatory cytokine differentially expressed in IL18 responsive cells. Since then, its expression has been implicated in various pathologies, including rheumatoid arthritis, pathogen responses, atherosclerosis, and several malignancies [3–12]. IL32 is broadly expressed in immune cells, including natural killer cells [13–15], T lymphocytes [16–18], macrophages, and dendritic cells [19, 20]. Functionally, IL32 can activate NF-κB and p38 mitogen activated protein kinase pathways and induce expression of proinflammatory cytokines including TNFα, IL8 and CCL2. In addition to its presence in immune tissues, IL32 expression can also be induced in human epithelial tissues. IL32 is also expressed in a broad range of human epithelial cancers–gastric [3], lung [5], breast [4], colon [6–9], pancreas [10], and thyroid [11, 12]—as well as hematologic malignancies such as lymphoma and leukemia [16, 21]. IL32 expression in cancer is linked to features associated with worse prognosis, including angiogenesis, invasion, and metastasis [4].

However, the role of IL32 in human melanoma cells is less well understood. Initial studies suggested that IL32 expression was related to a more invasive, metastatic phenotype marked by a loss of e-cadherin expression [22]. Others reported that IL32 isoforms alpha and gamma were highly enriched in PD-L1 expressing melanoma specimens [23].

Herein we report an unbiased analysis of IL32 expression in melanoma using RNA sequencing data from: [1] a large in-house panel of melanoma cell lines and [2] tumor tissues from The Cancer Genome Atlas (TCGA) dataset. IL32 expression correlated with a dedifferentiated phenotype which has been characterized by resistance to targeted therapies, escape from immune recognition, and a high AXL/low MITF genetic signature [24–27]. Expression of IL32 in human melanoma can be transcriptionally regulated in the context of a proinflammatory tumor microenvironment with either induced or constitutive IL32 expression strongly, but not invariably, correlating with a dedifferentiated genetic signature.

## Methods

### Cell culture

The human melanoma cell lines were established from patient biopsies. These cells were grown in RPMI 1640 media supplemented with 10% FBS, 1% l-glutamine and 1% penicillin/streptomycin/actinomycin D (Life Technologies, Grand Island, NY). Melanoma cell lines were treated with 1000 IU/mL TNFα and 100 IU/mL IFNγ (PeproTech US, Rocky Hill, NJ) for the indicated time points. M397 was cultured with recombinant 100 ng/mL IL32α, -β, or -γ (R&D Systems, Minneapolis, MN) for 7 days, with fresh IL32 being added every 2–3 days. Cells were counted using Trypan blue. Jurkat T lymphocytes were cultured in RPMI 1640 supplemented with 10% FBS.

### Gene expression analysis

Gene expression FPKM values for the melanoma cell line panel was obtained from the Gene Expression Omnibus (GEO) accession number GSE80829. The hallmark geneset for NF-κB and TNFα signaling (TNFA_SIGNALING_VIA_NFKB) was downloaded from the Molecular Signatures Database (MSigDB). Gene set enrichment analysis of the full ranked list of IL32 correlations was performed using the pre-ranked tool and the GO Biological Process Ontology gene sets from MSigDB v6.1. Gene expression FPKM values of skin cutaneous melanoma tumor biopsies from The Cancer Genome Atlas (TCGA SKCM) was downloaded from the Genomic Data Commons (portal.gdc.cancer.gov). For all analyses, FPKM values were log2 transformed with an offset of 1. Calculation of the enrichment overlap of the top 100 correlated genes with IL32 was performed using the MSigDB investigate gene sets tool (software.broadinstitute.org/gsea/msigdb/annotate.jsp) and the hallmark gene sets.

### Retroviral vectors

IL32 isoforms were cloned into an MSCV retroviral vector with a T2A-GFP using the HiFi DNA Assembly Cloning Kit (NEB, Ipswich, MA). The retroviral vector was kindly provided by A. Ribas. Retroviruses were produced using GP2-293 cells (Clontech, Mountain View, CA) and human melanoma cells were transduced with IL32 expressing retroviral vectors with 4ug/mL Polybrene. GFP positive cells were sorted 48 h after transduction. Cells maintained approximately 95% GFP positivity over the course of the experiment. IL32 expression was confirmed by real time PCR and Western blot.

### Real time PCR

RNA was extracted using PureLink RNA Mini Kit (ThermoFisher, Waltham, MA) and cDNA was synthesized using the High-Capacity RNA-to-cDNA kit (ThermoFisher, Waltham, MA). Real time PCRs were performed using the Applied Biosystems 7500 using the following primers designed using NCBI Primer-BLAST. GAPDH was used a housekeeping gene.

IL32 Primers:

IL32a Fwd: 5′-GAGGCAACAGATCCCCTGTC-3′.

IL32a Rev: 5′-GGCTCCGTAGGACTTGTCAC-3′.

IL32b Fwd: 5′-TCTCTCGGCTGAGTATTTGTGC-3′.

IL32b Rev: 5′-ATGACCCAGCTCCACTGAGA-3′.

IL32 g Fwd: 5′-TACTTCTGCTCAGGGGTTGG-3′.

IL32 g Rev: 5′-TGGGTGCTGCTCCTCATAAT-3′.

Differentiation gene set:

NGFR Fwd: 5′-TCATCCCTGTCTATTGCTCCA-3′.

NGFR Rev: 5′-TGTTCTGCTTGCAGCTGTTC-3′.

MLANA Fwd: 5′-GCTCATCGGCTGTTGGTATT-3′.

MLANA Rev: 3′-TTCTTGTGGGCATCTTCTTG-5′.

MITF Fwd: 5′-ATCAGCAACTCCTGTCCAGC-3′.

MITF Rev: 5′-GCCAGTGCTCTTGCTTCAGA-3′.

AXL Fwd: 5′-ACCTACTCTGGCTCCAGGATG-3′.

AXL Rev: 5′-CGCAGGAGAAAGAGGATGTC-3′.

GAPDH Fwd: 5′-TGCACCACCAACTGCTTAGC-3′.

GAPDH Rev: 5′-GGCATGGACTGTGGTCATGAG-3′.

### 5′ rapid amplification of cDNA ends (RACE)

To determine the transcriptional start site of IL32, 5′ RACE was performed according the manufacturer’s directions for the SMARTer RACE 5′/3′ kit (Takara, Mountain View, CA). Briefly, RNA was isolated from the IL32 expressing cell line, M318, using NucleoSpin RNA II kits (Takara, Mountain View, CA). First strand cDNA synthesis was done according the manufacturer’s directions. The 5′ RACE reaction using the following gene specific primer and a universal primer (Universal Primer Short, UPM, Takara) using 25 cycles of 94° for 30 s, 68° for 30 s, and 72° for 3 min. RACE products were run on an agarose gel and purified using the NucleoSpin Gel and PCR Clean-Up kit (Takara, Mountain View, CA). RACE products were cloned into the pRACE vector using the In-Fusion HD Cloning kit (Takara, Mountain View, CA) and sequenced using the M13F and M13R sequencing primers.

### Luciferase constructs

The promoter region of IL32 were pcr’d from the Human BAC clone RP11 (RPC1-11 clone 473M20) using Pfusion DNA polymerase (NEB, Ipswich, MA). Promoter region greater than 3 kb were cloned into pGL3 using HiFi DNA Assembly Cloning Kit (NEB, Ipswich, MA). The promoter regions were derived using the following primers.

− 10.5 kb Fwd: 5′-ACCGAGCTCTTACGCGTGCTAGCCCGCAGGGAAGAAGGTGAGAGATG-3′.

− 7.5 kb Fwd: 5′-ACCGAGCTCTTACGCGTGCTAGCCCCTCTTTAGCGGTGAGTGGGG-3′.

− 5.5 kb Fwd: 5′-ACCGAGCTCTTACGCGTGCTAGCCCCAGTAGCTGGCTGTTTCGTG-3′.

− 2.5 kb Fwd: 5′-CTAGCCCGGGCTCGAGATCTAGAAAGTAGATGAGGCCAG-3′.

− 2 kb Fwd: 5′-CTAGCCCGGGCTCGAGATCTAAAACAGGGTACATACAGTCTG-3′.

− 1.5 kb Fwd: 5′-CTAGCCCGGGCTCGAGATCTGGAGTGCAGTGGCACCAT-3′.

− 1.0 kb Fwd: 5′-CTAGCCCGGGCTCGAGATCTGTTTGGCCCCAGGAAACC-3′.

− 0.5 kb Fwd: 5′-CTAGCCCGGGCTCGAGATCTCCCCAGCCAGCTGTCCCG-3′.

− 2.5 kb to − 0.5 kb Rev: 5′-CTCGAGCTCGAGTGGCGGCCAAAAGTTCAAGGAGC-3′.

− 10.5 kb to − 3 kb Rev: 5′-GAATGCCAAGCTTACTTAGATCGCATGGCGGCCAAAAGTTCAAG-3′.

Promoter regions were cloned into the SmaI/XhoI (less than − 3.0 kb) or HindIII (greater than − 3.0 kb) sites of pGL3 Luciferase reporter vector (Promega, Madison, WI). Promoter constructs were confirmed by Sanger sequencing. Promoter constructs were transfected into 1.8–2 × 10^4^ cells human melanoma cells in equal molar concentrations using Lipofectamine LTX with PLUS Reagent (ThermoFisher, Waltham, MA). pGL4.74 (hRluc/TK, Promega, Madison, WI) was used for transfection normalization. Firefly and Renilla luciferase expression was assessed 48 h after transfection using the Dual-Glo Luciferase Assay system (Promega, Madison, WI). Cells were treated with 1000 IU/mL TNFα and 100 IU/mL IFNγ (PeproTech US, Rocky Hill, NJ) 24 h prior to assessing luciferase activity. All values were normalized to the pGL3 empty vector control.

### Myeloid cell polarization

PBMCs were collected using a ficoll gradient (Ficoll-Paque Plus, GE Healthcare) and monocytes were isolated by a CD14 positive selection (Miltenyi, Bergisch Gladbach, Germany). Monocytes were split into five experimental groups: (1) Negative Control (no cytokines), (2) GM–CSF (Sanofi) + IL-4 (Cell Genix) at 1000 U/ml, 3) Recombinant IL32α (R&D Systems) at 100 ng/ml, 4) Recombinant IL32β (R&D Systems) at 100 ng/ml, 5) Recombinant IL32γ (R&D Systems) at 100 ng/ml, and cultured using Cell Genix Media to yield immature DCs at day 5. Immature DC were harvested and surface stained for flow cytometry analysis. Cell surface markers were observed on the double positive, HLA-DR and CD86 population of cells. Antibodies used included CD80 FITC (BD, Clone L307.4), Mouse IgG1 FITC (Beckman Coulter PN IM0639U), CD86 Pe-Cy7 (BD, Clone FUN-1), HLA-DR PerCpCy5.5 (BD, Clone G46-4), CD1B APC (BioLegend, Clone SN13), CD14 APC-Cy 7 (BD, Clone MφP9), CD68 BV 711 (BD, Clone Y1/82A), Mouse IgG2B BV 711 (BD, Clone 27-35), and Zombie Aqua Viability Dye BV 510 (BioLegend).

### Statistical analyses

Gene expression bar graphs are shown as mean and standard differentiation. Gene expression was normalized to GAPDH and expressed as Delta Ct values. Comparison between the reference control (untreated M397, M398, and M249) was performed with one-way analysis of variance (ANOVA, 95% CI) with a Dunnetts’ multiple comparisons test. All calculations were done using Prism software. p-values less than 0.05 were considered statistically significant. For luciferase activity, data was normalized to Renilla luciferase expression and fold changes were calculated against the pGL3 empty control vector. Error bars represent standard deviation.

## Results

### IL32 expression in human melanoma

**Fig. 1:**
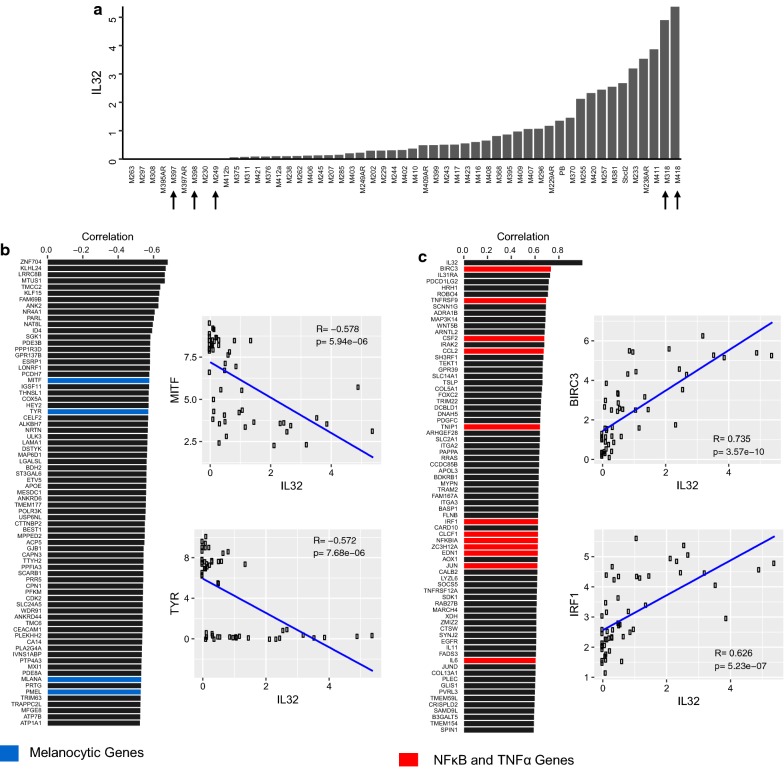
IL32 is expressed in dedifferentiated melanomas. **a** Bar plot of log2 FPKM expression values across of panel of melanoma cell lines. **b** Bar plot showing the top 75 genes most correlated with IL32 expression. Genes highlighted in blue are melanocytic genes. (left) Scatterplot of log2 FPKM expression values between select melanocytic genes and IL32 (right). **c** Bar plot showing the top 75 genes most anti-correlated with IL32 expression. Genes highlighted in red are TNFα signaling and NF-κB associated genes. (left) Scatterplot of log2 FPKM expression values between select anti-correlated genes and IL32 (right)

We examined RNA sequencing data from a panel of 53 established human melanoma cell lines derived from resected metastatic deposits [25, 26]. Across this dataset, IL32 expression was detectable in a majority of the melanoma cell lines (Fig. 1a). IL32 expression in a subset of melanoma cell lines was confirmed by real time PCR (data not shown). Immunohistochemical staining of IL32 in melanoma tissues, pulled from The Human Protein Atlas, indicates a range of expression within tumor samples (Additional file 1: Figure S1A) [28, 29]. However, it is notable that IL32 levels in melanoma are low relative to other tumor cell lines represented in the NCI-60 Cancer Cell Line database or the Cancer Cell Line Encyclopedia (Additional file 1: Figures S1B, C, respectively) [30, 31].

### IL32 expression in human melanoma cell lines and tissues correlates with a dedifferentiated gene signature

We globally assessed IL32 co-expression patterns across the 53 cell lines by calculating the Pearson correlation between the expression of IL32 to all genes in the dataset. Among the top 75 anti-correlated genes with IL32 expression, we found classical melanocyte and pigment associated genes such as *MITF*, *TYR, PMEL*, and *MLANA* (Fig. 1b). Gene expression scatterplots of MITF and TYR vs. IL32 show that melanoma cells with high IL32 expression are typically dedifferentiated, whereas differentiated melanoma are associated with absent or lower levels of IL32 (Fig. 1b). Furthermore, among the top 20 differentially expressed genes in melanoma cell lines in the lowest quartile of IL32 expression (compared to top quartile), we identified enrichment of genes related to pigmentation and melanogenesis (*TYR, MLANA, PMEL, TYRP1, DCT, and MITF;* hypergeometric p-value = 7.9 × 10^−15^).

Previous studies have shown that a dedifferentiated MITF-low transcriptional state is associated with an NF-κB-high inflammatory cell state and that TNFα treatment can promote dedifferentiation [24, 27, 32]. Therefore, we investigated if genes positively correlated to IL32 were involved in NF-κB or TNFα signaling. To assess this, we obtained NF-κB and TNFα signaling genes from the well-curated hallmark collection [33]. Within the top 75 genes highly correlated to IL32 expression, we observed a strong enrichment for the NF-κB and TNFα hallmark genes (hypergeometric p-value = 2.1 × 10^−11^) (Fig. 1c). Scatterplots for representative genes are shown in Fig. 1c. TNFα and IL32 interactions have previously been described in the literature [16].

As a confirmatory step, we performed Gene Set Enrichment Analysis on the full ranked list of IL32 expression correlations. Supportive of our earlier findings, the top gene sets highly enriched for IL32 anti-correlation include pigmentation and metabolic gene sets, and the top most highly enriched for IL32 expression include NF-κB and immune related gene sets (Tables 1, 2). Together, this suggests that IL32 is among several hundred melanoma genes whose expression is affected by exposure to these proinflammatory cytokines.

**Table 1:** Gene set enrichment analysis (GSEA) of genes correlated with IL32

Name	NES	p-value	FDR q-value
1. Go I kappab kinase NF kappab signaling	2.461	< 0.001	< 0.001
2. Go cellular response to mechanical stimulus	2.456	< 0.001	< 0.001
3. Go positive regulation of chemotaxis	2.302	< 0.001	2.008E−03
4. Go tumor necrosis factor mediated signaling pathway	2.286	< 0.001	1.506E−03
5. Go modification by symbiont of host morphology or physiology	2.266	< 0.001	1.449E−03
6. Go response to mechanical stimulus	2.257	< 0.001	1.601E−03
7. Go positive regulation of response to external stimulus	2.223	< 0.001	2.745E−03
8. Go toll like receptor signaling pathway	2.217	< 0.001	2.698E−03
9. Go pattern recognition receptor signaling pathway	2.215	< 0.001	2.533E−03
10. Go NIK NF kappab signaling	2.212	< 0.001	2.280E−03

Top ten enriched gene sets correlated with IL32 include NF-κB and immune-related gene sets

**Table 2:** Gene set enrichment analysis (GSEA) of genes anti-correlated with IL32

Name	NES	p-value	FDR q-value
1. Go developmental pigmentation	− 2.619	< 0.001	< 0.001
2. Go melanocyte differentiation	− 2.500	< 0.001	< 0.001
3. Go cellular respiration	− 2.456	< 0.001	< 0.001
4. Go mitochondrial respiratory chain complex assembly	− 2.446	< 0.001	< 0.001
5. Go oxidative phosphorylation	− 2.400	< 0.001	< 0.001
6. Go pigmentation	− 2.377	< 0.001	< 0.001
7. Go pigment cell differentiation	− 2.374	< 0.001	< 0.001
8. Go aerobic respiration	− 2.347	< 0.001	1.83E−04
9. Go tricarboxylic acid metabolic process	− 2.251	< 0.001	6.51E−04
10. Go mitochondrial respiratory chain complex I biogenesis	− 2.228	< 0.001	8.04E−04

Top ten enriched gene sets negatively correlated with IL32 include pigmentation and metabolic gene sets

### TNFα and IFNγ induced IL32 expression in general synchrony with dedifferentiation in melanoma cell lines

**Fig. 2:**
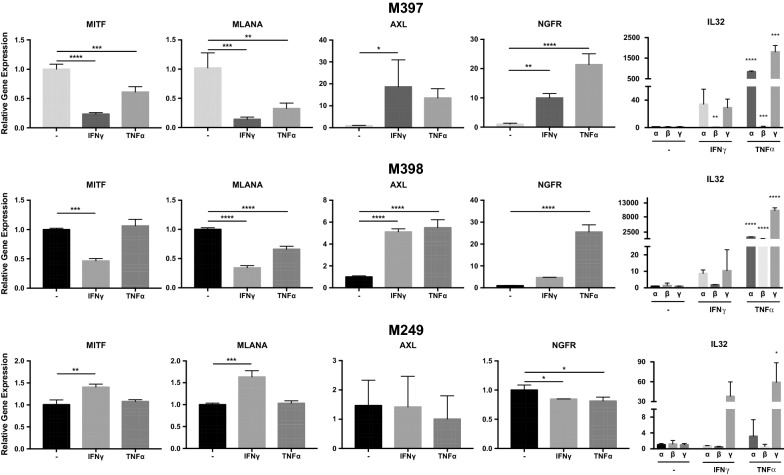
TNFα and IFNγ treatment on melanoma cell lines results in dedifferentiation and IL32 gene expression. M397, M398, and M249 melanoma cell lines were treated with 1000U/mL TNFα or 100 U/mL IFNγ for 3 days and changes in gene expression were assessed by real time PCR. Gene expression for each sample was normalized to GAPDH and expressed as Delta Ct values, with the untreated M397, M398, M249 as the reference control. Error bars, standard deviation (**p < *0.01*, ***p < *0.001*, 95% CI, 1-way ANOVA). Figure is a representative experiment from 3 replicate experiments

**Fig. 3:**
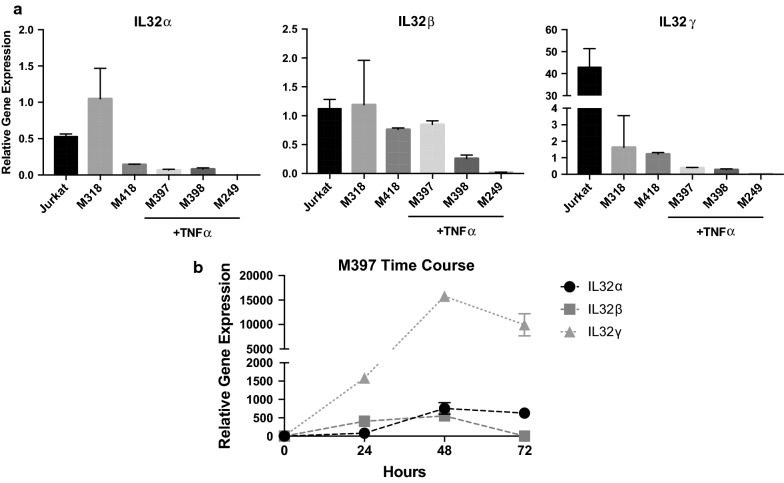
Level of IL32 isoform expression after TNFα treatment. **a** IL32 gene expression (α, β, γ) after 3 day treatment with 1000 U/mL TNFα was compared to two IL32 expressing melanoma cell lines (M318 and M418) and Jurkat cells. Gene expression was normalized to GAPDH and expressed as Delta Ct values, with the Jurkat cells as the reference control. **b** Induction of IL32 gene expression with 24, 48, and 72 h treatment with 1000 U/mL TNFα. Gene expression was normalized to GAPDH and Delta Ct values are compared to the day 0 reference control

To evaluate if IL32 is induced with inflammatory signaling, three non-IL32-producing melanoma cell lines (M397, M398, M249) were exposed to TNFα or IFNγ for 72 h (Fig. 2). We selected these three melanoma cell lines to observe the parallel impact of TNFα and IFNγ on IL32 and the melanoma differentiation state because they exhibited a low AXL/high MITF differentiated genetic signature. Both TNFα and IFNγ induced expression of the three predominant isoforms of IL32 (α, β, γ) in all three cell lines at varying levels. Overall, the impact of TNFα on IL32 expression was greater than the impact of IFNγ. Induction of IL32γ by TNFα was the most notable response in all three cell lines. The relative level of IL32 isoform transcripts in these three cell lines, after TNFα induction, is generally lower than the constitutive expression in the two high IL32-producing, dedifferentiated cell lines M318 and M418 (Fig. 3a). For reference, we also evaluated IL32 transcript levels in the Jurkat T cell line, a recognized high IL32 expressing leukemia cell line. In general, with the exception of M318, the γ isoform is the most abundant and when induced by cytokine stimulation has significantly higher and more prolonged induction (Figs. 2, 3a, b).

To test the parallel impact of IL32 induction with TNFα or IFNγ treatment on differentiation, we also evaluated expression of melanocytic markers *MITF* and *MLANA*, neural crest marker *NGFR* and the receptor tyrosine kinase *AXL*. A large and growing body of literature has shown the expression of AXL strongly inversely correlated with the expression of MITF, with a high AXL/MITF ratio associated with therapy resistance [24, 25]. At baseline, these three melanoma cell lines exhibited a low AXL/high MITF differentiated genetic signature. In two of three cell lines (M397 and M398) we observed inversion of the AXL/MITF ratio towards a dedifferentiated genotype in parallel with induction of IL32. However, one of three cell lines (M249) did not dedifferentiate in response to TNFα or IFNγ, despite induction of IL32. The lack of correlation between IL32 induction and inverted AXL/MITF ratio in M249 suggests that one event is not invariably accompanied by the other. Furthermore, enforced expression of recombinant IL32α, -β, or -γ in M397 did not impact expression of melanoma differentiation genes *MITF, AXL, NGFR or MLANA*, strongly suggesting that IL32 is not a causal or upstream event in the melanoma dedifferentiation process (Additional file 2: Figure S2).

### Proinflammatory cytokines TNFα and IFNγ induce IL32 promoter activity in melanoma cell lines

**Fig. 4:**
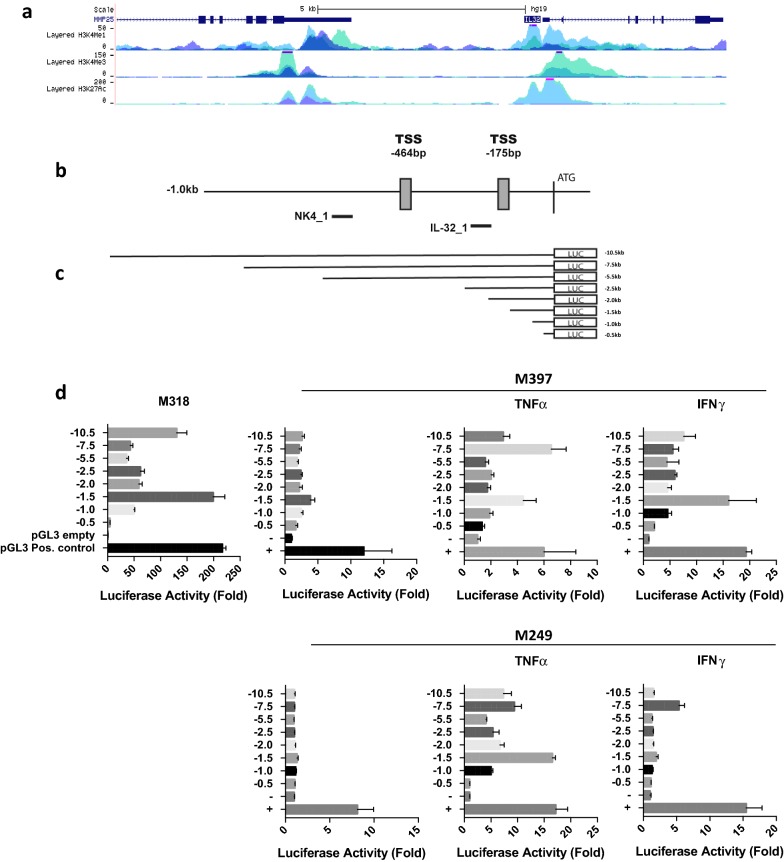
IL32 Promoter Activity. **a** Encode data set, **b** Promoter region of IL32. TSS determined from 5′RACE are indicated at − 464 bp and − 175 bp from the ATG. Two promoter sites identified by The Eukaryotic Promoter Database are identified as NK4_1 and IL32_1. **c** Promoter constructs used in luciferase assays. **d** Promoter constructs were co-transfected with pGL4.73 (Renilla) into melanoma cell lines (M318, M397, and M249). Post-transduction cells were treated for 24 h with 1000 U/mL TNFα and 100 U/mL IFNγ. Luciferase activity was assessed using Dual-Glo according to the manufactures directions. Data was normalized to Renilla luciferase expression and fold changes were calculated against the pGL3 empty control vector. Luciferase assays were performed in triplicate and plotted data is a representative experiment of three independent experiments

We characterized the transcriptional regulation of IL32 in human melanoma, both in constitutive and cytokine-induced cells. CHIP-seq analysis of active enhancer and promoter elements, as defined by H3K4Me1, H3K27Ac, H3K3Me3 marks [34–37] in HUVEC and K562, cell lines known to express IL32, indicate the presence of regulatory elements 5′ relative to the IL32 translational start site (ATG) (Fig. 4a, ENCODE data set; University of California, Santa Cruz (UCSC) genome browser). Using 5′ RACE we determined the location of the transcription start site (TSS) in the IL32 positive cell line M318; two TSS were identified at − 464 bp and − 175 bp (chr16: 3,115,349 and chr16: 3,115,638) relative to the ATG (Fig. 4b). The Eukaryotic Promoter Database (EPD; http://epd.vital.it.ch) identified two putative IL32 promoters EPD NK4_1 and EPD IL32_1, which are − 558bps and − 221bps relative to the ATG (see “Discussion” Fig. 4b). Based on this information, we cloned various lengths of the IL32 5′ upstream genomic region (− 10.5, − 7.5, − 5.5, − 2.5, − 2, − 1.5, − 1, − 0.5) into a luciferase reporter construct; these constructs encompassed the putative EPD promoters, both identified TSS sites, and the upstream (5′) ENCODE histone marks. These constructs were transiently transfected into IL32 negative (M249, M397) and IL32 positive (M318) cell lines (Fig. 4d). Strong promoter activity was noted in the IL32 positive but not the negative cell lines, with the highest level in the − 10.5 and − 1.5 constructs. When both IL32 negative cell lines were exposed to TNFα or IFNγ for 24 h, there was a significant induction of promoter activity. TNFα consistently induced stronger IL32 promoter activity than IFNγ. M249, which cannot be dedifferentiated with TNFα or IFNγ, also showed strong IL32 promoter induction consistent with IL32 transcript expression. The promoter construct profiles were similar in both constitutive and induced settings.

### IL32 in the melanoma microenvironment is associated with immune infiltration and melanoma dedifferentiation

Given the impact of TNFα and IFNγ on IL32 expression in melanoma cell lines, we investigated the expression of IL32 in the chemokine-rich melanoma tumor microenvironment using RNA sequencing data from tumor biopsies as part of The Cancer Genome Atlas (TCGA) dataset. We observed strong Pearson correlation coefficients between IL32 expression, found within the melanoma tumor and the surrounding microenvironment, and genes related to immune cell infiltration, such as CD3E (0.94), CD8A (0.89), IFNγ (0.75), and GZMB (0.86) (Additional file 3: Figure S3). Additionally, we observed a strong correlation between IL32 expression and several checkpoint receptors and ligands, such as PD-1 (0.87), PD-L1 (0.69), Tim-3 (0.82), Lag3 (0.85), and TIGIT (0.86) (Additional file 4: Figure S4). We also observed correlations between IL32 and genes related to other immune subsets, including myeloid cells (ITGAM, 0.71; CD68, 0.61; CD14, 0.74), dendritic cells (ITGAX, 0.71; ITGAE, 0.47) and B cells (CD19, 0.67). Consistent with this, the top 100 genes that correlated with IL32 expression in the TCGA dataset were enriched with gene sets related to allograft rejection (hypergeometric p-value = 4.3 × 10^−36^), inflammatory response (hypergeometric p-value = 3.3 × 10^−10^), and IFNγ signaling (hypergeometric p-value = 8.3 × 10^−9^).

In light of the impact of a proinflammatory microenvironment on melanoma differentiation, as well as the association between IL32 and dedifferentiation in melanoma cell lines, we investigated if this phenomenon was also present in the TCGA dataset. We observed a correlation between IL32 expression in melanoma tumor biopsies with a high AXL/low MITF drug-resistant signature, consistent with data from melanoma cell lines (Additional file 3: Figure S3). However, IL32 did not inversely correlate with signature melanocyte genes (*MLANA, PMEL, TYR*) as we observed in the cell line data (data not shown). Additionally, IL32 expression did not correlate with genes associated with disease progression, tumor cell invasion, and migration, such as *CD155/PRV* and *MMP2* (Additional file 4: Figure S4).

### IL32 isoforms have differential impact on myeloid polarization

IL32 from melanoma cells may influence non-melanoma cells in the tumor microenvironment, such as myeloid cells, which can play both pro- and anti-tumor roles depending on their phenotypic state. We preliminarily assessed of the impact of IL32 on the polarization of myeloid cells. It has previously been reported that IL32γ induces the differentiation of monocytes into phagocytic macrophage-like cells (though they discordantly express CD14 and CD1a) [19]. However, the effect of IL32γ, or other IL32 isoforms (IL32α and IL32β), on myeloid cell polarization is not well understood. We observed that monocytes cultured with either IL32β or IL32γ express both CD68 and CD80, markers associated with M1 proinflammatory macrophages thought to play an anti-tumor role (Additional file 5: Figure S5). In contrast, exposure to IL-32α resulted in a CD68+ but CD80− population. Likewise, IL32β and IL32γ, but not IL32α, resulted in higher CD14 and CD1b expression.

## Discussion

Human melanomas produce a number of cytokines generally associated with the immune system [38]. In an analysis of the RNA transcripts from 53 established melanoma lines, a significant proportion express IL32 isoforms. Thus, we evaluated the biology underlying IL32 expression in the context of melanoma both in cell lines and in melanoma tumor samples.

IL32 is found on human chromosome 16p13.3, is expressed in at least 6 splice variants (α, β, γ, δ, ε, ζ) and was originally isolated from activated natural killer and T cells [16]. Which isoforms are secreted as opposed to acting intracellularly is still unresolved and hampered by an as of yet unidentified cell surface receptor [39, 40]. The γ isoform is the only isoform that has a leader sequence, is composed of all exons, is believed to be secreted, and believed to be the most biologically active [41]. A rodent IL32 homologue does not exist-raising the obvious question as to whether or not this gene product is dispensable or redundant-but human recombinant and transgenic IL32 appears to be biologically active in mice [12, 42–44]. Despite these gaps in our knowledge, much of the published literature on the biology of IL32 comes from experiments utilizing commercially available recombinant protein, generally the α, β, and γ isoforms [1].

Several themes emerge regarding IL32 biology: (a) IL32 can modulate the production of various cytokines including IL1β [45], TNFα [46], IL6 [47], IL8 [8], and may activate cells of the immune system [15, 19]; (b) IL32 expression can be induced by various viruses and microbes [2, 20, 42, 48–51] and may play a role in antiviral immunity [52]; (c) IL32 may have direct effects on various cancer cells in vitro; [8, 9, 22, 47, 53, 54]; (d) IL32 may play a role in tumor immunity [4, 7, 12, 14, 44]; [41]; and (e) IL32 has been associated with various inflammatory diseases [43, 45], such as ulcerative colitis and rheumatoid arthritis [55]. It is also curious that, largely from immunohistochemistry studies, IL32 is expressed by a broad range of epithelial and in hematopoietic malignancies and is generally correlated with aggressive biology, although no unifying mechanism has been identified.

In comparing IL32 expressing and non-expressing melanoma cell lines, we observed that IL32 expression in melanoma cell lines correlates with a high AXL/low MITF ratio, a genetic signature which has been reported to be associated with a treatment-resistant, dedifferentiation “invasive” phenotype. This dedifferentiation phenotype results in reduced expression of melanocytic-lineage antigens, which may also provide a mechanism for immune evasion [32]. Melanomas, which progress on MAPK inhibitor therapies, acquire this genetic signature of reduced MITF and RTK upregulation [25]. MITF controls the expression of a broad range of genes in melanocyte-lineage cells that govern differentiation, migration, and proliferation [56–58]. The low MITF signature is regulated by receptor tyrosine kinases, including AXL [59].

The plasticity of these neural crest-derived cutaneous malignancies is underscored by the ability of an inflammatory signal—TNFα or IFNγ, for example—to effect a differentiated to dedifferentiated conversion in melanoma cell lines with only a 72 h in vitro exposure, a phenomenon that reverses when the cytokine is removed from culture [26]. We found that TNFα and IFNγ, which promote a dedifferentiated melanoma phenotype, also induce IL32 expression in non-IL32 expressing melanoma cell lines by impacting activity at the promoter level.

Palstra et al. recently reported that the DNA element encompassing rs4349147 is a strong long distance enhancer essential for the expression of IL32 in CD4 T cells located 10 kb 3′ of the IL32 promoter [52]. Jurkat cells are heterozygous for the rs4349147-A and rs4349147-G alleles. The A allele increases the expression of IL32α, generally viewed as anti-inflammatory, whereas the G allele promoted the expression of the proinflammatory IL32γ and IL32β isoforms. These proinflammatory isoforms enhanced lymphocyte activation and susceptibility to HIV infection. This report provides considerable insight into various observations on the biology of IL32 isoforms. Two putative IL32 promoters have been identified using the Eukaryotic Promoter Database (http://epd.vital-it.ch), which are depicted in Fig. 4. These two promoters, at least in Jurkat cells, designated EPD NK4_1 and EPD IL32_1, are thought to support the transcription of IL32γ and IL32α/β, respectively.

Our IL32 promoter constructs encompass both the identified EPD NK4_1 and EPD IL32_1 putative promoter regions and extend 10.5 kb 5′ to the ATG. The luciferase activity driven by these constructs would then necessarily reflect constitutive and induced gene expression of all three isoforms, as well as the putative *cis*-acting elements residing within this 5′ upstream region. Palstra et al. performed formaldehyde-assisted isolation of regulatory elements (FAIRE) assays in Jurkat and a melanoma cell line (G361), which demonstrated significant increase in DNA accessibility in the region surrounding rs4349147 in the former but not the latter cell line [52]. We therefore conjecture that this long-range enhancer may not play a role in melanoma IL32 transcription. In another IL32 promoter study using Akt-activated endothelial cell line constructs extending 2.5 kb 5′ to the ATG [60]; the difference in cell lines and activation signals precludes any meaningful comparison.

Given the impact of proinflammatory cytokines on IL32 expression, we investigated the IL32 expression in immunologically rich melanoma tumors using RNA sequencing data available in the TCGA dataset. IL32 expression was highly correlated with a T cell dominant immune signature in these tumors, probably a result of immune cell IL32 production. We also observed a correlation between IL32 expression, derived from tumor cells and the surrounding microenvironment, and a high AXL/low MITF gene signature, as in the melanoma cell lines. However, we interpret this result cautiously because, unlike in the melanoma cell line dataset, IL32 expression in the melanoma tumor microenvironment did not inversely correlate with pigmentation or melanoma differentiation genes such as *MLANA, TYR* or *PMEL*. Thus, the high AXL/low MITF signature may be a result of increased IL32 expression by infiltrating immune cells, rather than IL32 expressing dedifferentiated melanoma cells in the tumor microenvironment.

There is substantial evidence that IL32 induces dedifferentiation of human monocytes towards a macrophage-like phonotype with dendritic cell-like aspects. IL32 exposed monocytes altered their morphology within 3 days (flattening with extensive pseudopodia), which was accompanied by increased expression of CD1 and CD14 [19]. These macrophage-like cells exhibited active phagocytic properties and were also induced to express TNFα, IL-1β and IL6. Our preliminary in vitro studies indicated that IL32, in an IL32β and IL32γ isoform specific manner, can modulate the polarization of myeloid cells toward an M1-like macrophage phenotype with costimulatory molecule expression, lending additional support for its impact in the tumor microenvironment. It is plausible, then, that IL32 is expanded within the microenvironment as a byproduct of the feedback loop between the anti-tumor inflammatory response (marked by TNFα and IFNγ) and dedifferentiation of melanoma [23].

## Conclusions

In summary, this study shows that a significant percentage of cell lines derived from metastatic melanoma express IL32. IL32 expression, which correlates with a dedifferentiated “invasive” genetic signature, can be induced by inflammatory molecules TNFα and IFNγ that modulate IL32 transcriptional activity at the promoter level. IL32 expression by dedifferentiated tumor cells may contribute to a proinflammatory tumor microenvironment.

The remarkable advances in melanoma immunotherapy occasioned by the development of CTLA-4 and PD-1/PDL1 checkpoint inhibition underscores the importance of understanding potential contributions of melanoma-elaborated cytokines such as IL32 [61].

## Additional files

**Additional file 1: Figure S1.** Expression of IL32 in human melanoma. (A) Immunohistochemistry analysis of IL32 expression in cutaneous melanoma in two patients with low and high IL32 expression, respectively (images and data acquired from The Human Protein Atlas, and Image available at https://www.proteinatlas.org/ENSG00000008517-IL32/pathology/tissue/melanoma#img). Left panel is a cutaneous melanoma sample from a 73-year old woman (patient id: 2900). Right panel is a cutaneous melanoma sample from an 83-year old man (patient id: 2156). Staining was performed using BioLegend mouse anti-human IL32 monoclonal antibody (Cat # 513401) at a 1:4500 dilution after HIER antigen retrieval (pH = 6). (B and C) IL32 transcript expression across multiple different cancer lines organized by cancer type from the NCI-60 Cancer Cell Line database and the Cancer Cell Line Encyclopedia, respectively.

**Additional file 2: Figure S2.** Addition of recombinant IL32 does not impact melanoma cell line growth or differentiation. (A) Tumor growth over time in parental M397 melanoma cell line, compared to M397 treated with recombinant IL32α, -β or -γ. (B and C) Expression of melanoma differentiation genes by quantitative RT-PCR at baseline or after treatment with TNFα, recombinant IL32α, IL32β, or IL32γ measured at day 3 (B) or day 7 (C).

**Additional file 3: Figure S3.** IL32 expression in the TCGA dataset. (A-B) Scatterplot of log2 FPKM expression values between IL32 and select immune genes (A) or between the ratio of AXL and MITF log2 FPKM expression values (B) in the melanoma TCGA dataset (n = 479).

**Additional file 4: Figure S4.** IL32, checkpoint receptors/ligand expression, and markers of disease progression in the TCGA dataset. Scatterplot of log2 FPKM expression values between IL32 and select checkpoint receptors/ligands, as well as, select markers of disease progression (MMP2 and PVR).

**Additional file 5: Figure S5.** Expression of phenotypic markers on human monocytes after exposure to stimuli. CD14 + cells, from PBMC isolation, were cultured in the presence of GM-CSF + IL-4, Cell Genix Media, recIL32α, recIL32β, or recIL32γ for 5 days. The different treatments are displayed by using a gray scale. On day 5, the phenotype of the cells was assayed using flow cytometry analysis for various surface markers. GM-CSF + IL-4 and Cell Genix Media alone were as a positive and negative control, respectively. Cells were then assessed for surface expression of CD68, CD80, CD14, CD1B by flow cytometry. Frequency of positive cells is shown in the left panels, and mean fluorescence intensity is shown in the right panels. Statistical analysis was done using a one-way repeated measures ANOVA, using the GM-CSF + IL-4 treatment as a control. Each shape (circle, square, triangle) represents a different healthy donor. *P ≤ 0.05, **P ≤ 0.01, ***P ≤ 0.001.

## Abbreviations

IL32: interleukin 32
TCGA: the cancer genome atlas
ATG: translational start site
RACE: rapid amplification of cDNA ends
