# Supplementary figures and images for: Interleukin 32 expression in human melanoma
Source: J Transl Med. 2019 Apr 5;17:113. doi: 10.1186/s12967-019-1862-y (PMC6449995; doi:10.1186/s12967-019-1862-y)

## Slide 1
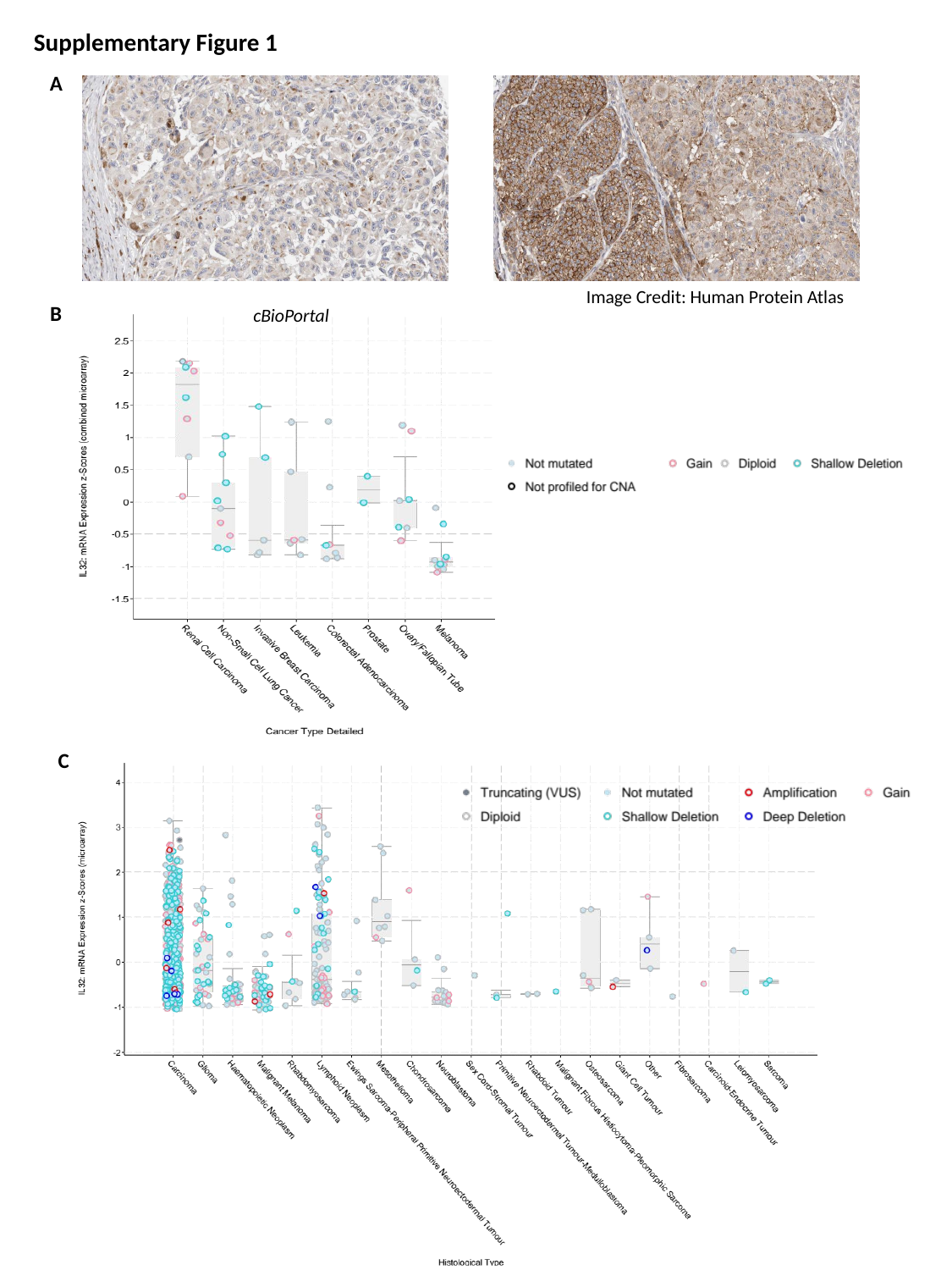

Supplementary Figure 1
A
Image Credit: Human Protein Atlas
B
cBioPortal
C

Supplement: Supplementary file 1 — Additional file 1: Figure S1. Expression of IL32 in human melanoma. (A) Immunohistochemistry analysis of IL32 expression in cutaneous melanoma in two patients with low and high IL32 expression, respectively (images and data acquired from The Human Protein Atlas, and Image available at https://www.proteinatlas.org/ENSG00000008517-IL32/pathology/tissue/melanoma#img). Left panel is a cutaneous melanoma sample from a 73-year old woman (patient id: 2900). Right panel is a cutaneous melanoma sample from an 83-year old man (patient id: 2156). Staining was performed using BioLegend mouse anti-human IL32 monoclonal antibody (Cat # 513401) at a 1:4500 dilution after HIER antigen retrieval (pH = 6). (B and C) IL32 transcript expression across multiple different cancer lines organized by cancer type from the NCI-60 Cancer Cell Line database and the Cancer Cell Line Encyclopedia, respectively. [file 12967_2019_1862_MOESM1_ESM.pptx]

## Slide 1
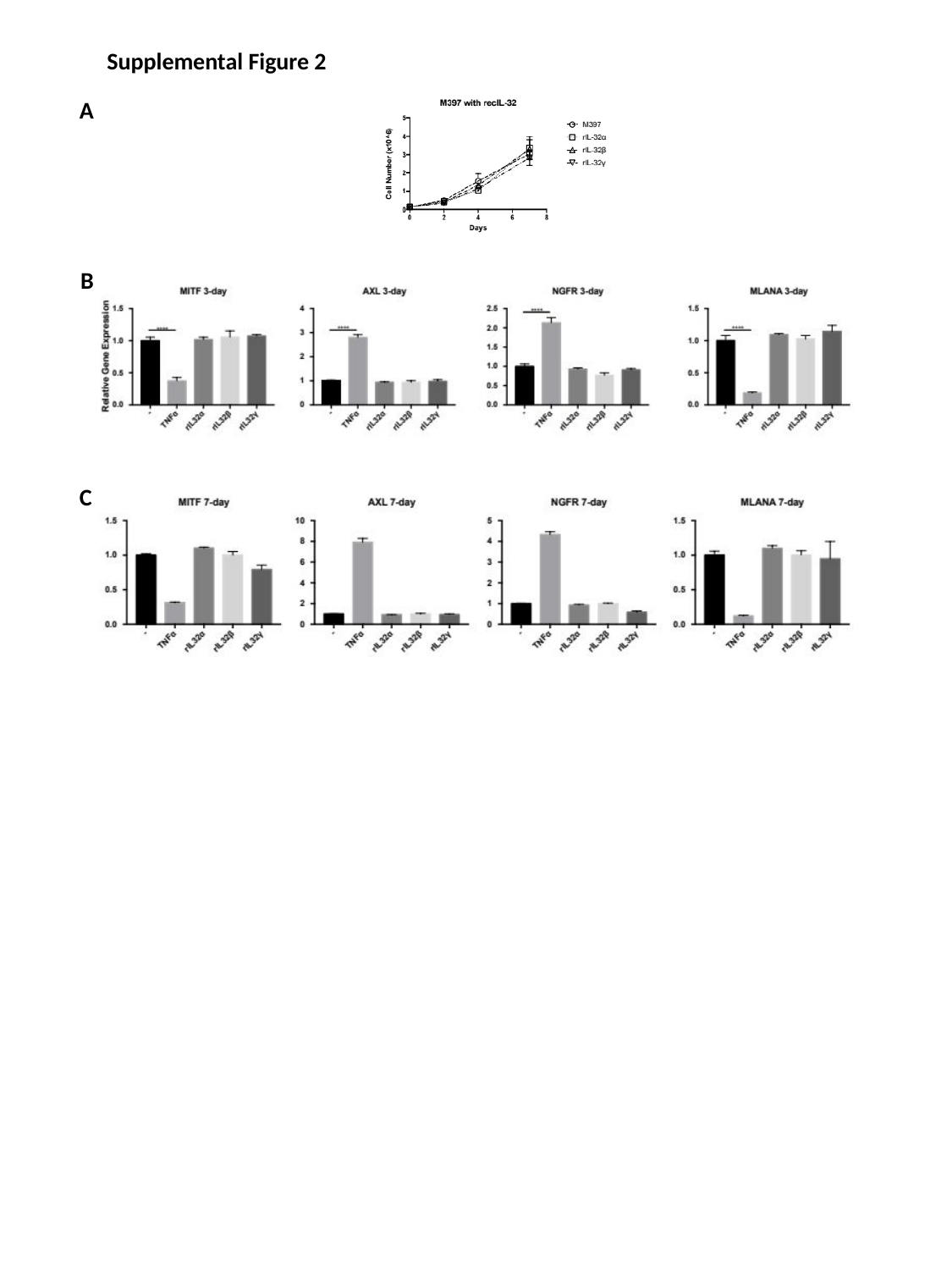

Supplemental Figure 2
A
B
C

Supplement: Supplementary file 2 — Additional file 2: Figure S2. Addition of recombinant IL32 does not impact melanoma cell line growth or differentiation. (A) Tumor growth over time in parental M397 melanoma cell line, compared to M397 treated with recombinant IL32α, -β or -γ. (B and C) Expression of melanoma differentiation genes by quantitative RT-PCR at baseline or after treatment with TNFα, recombinant IL32α, IL32β, or IL32γ measured at day 3 (B) or day 7 (C). [file 12967_2019_1862_MOESM2_ESM.pptx]

## Slide 1
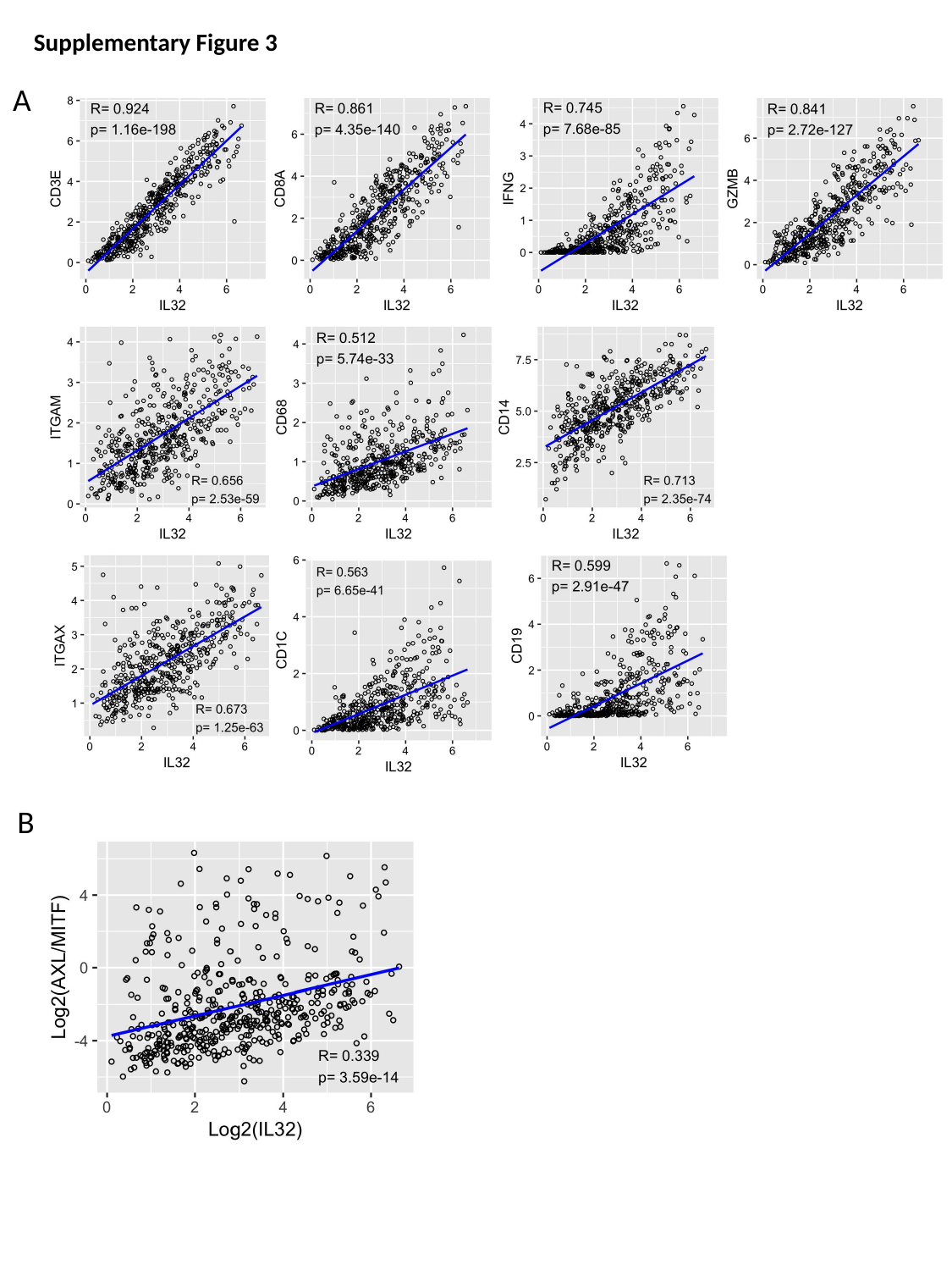

Supplementary Figure 3
A
B

Supplement: Supplementary file 3 — Additional file 3: Figure S3. IL32 expression in the TCGA dataset. (A-B) Scatterplot of log2 FPKM expression values between IL32 and select immune genes (A) or between the ratio of AXL and MITF log2 FPKM expression values (B) in the melanoma TCGA dataset (n = 479). [file 12967_2019_1862_MOESM3_ESM.pptx]

## Slide 1
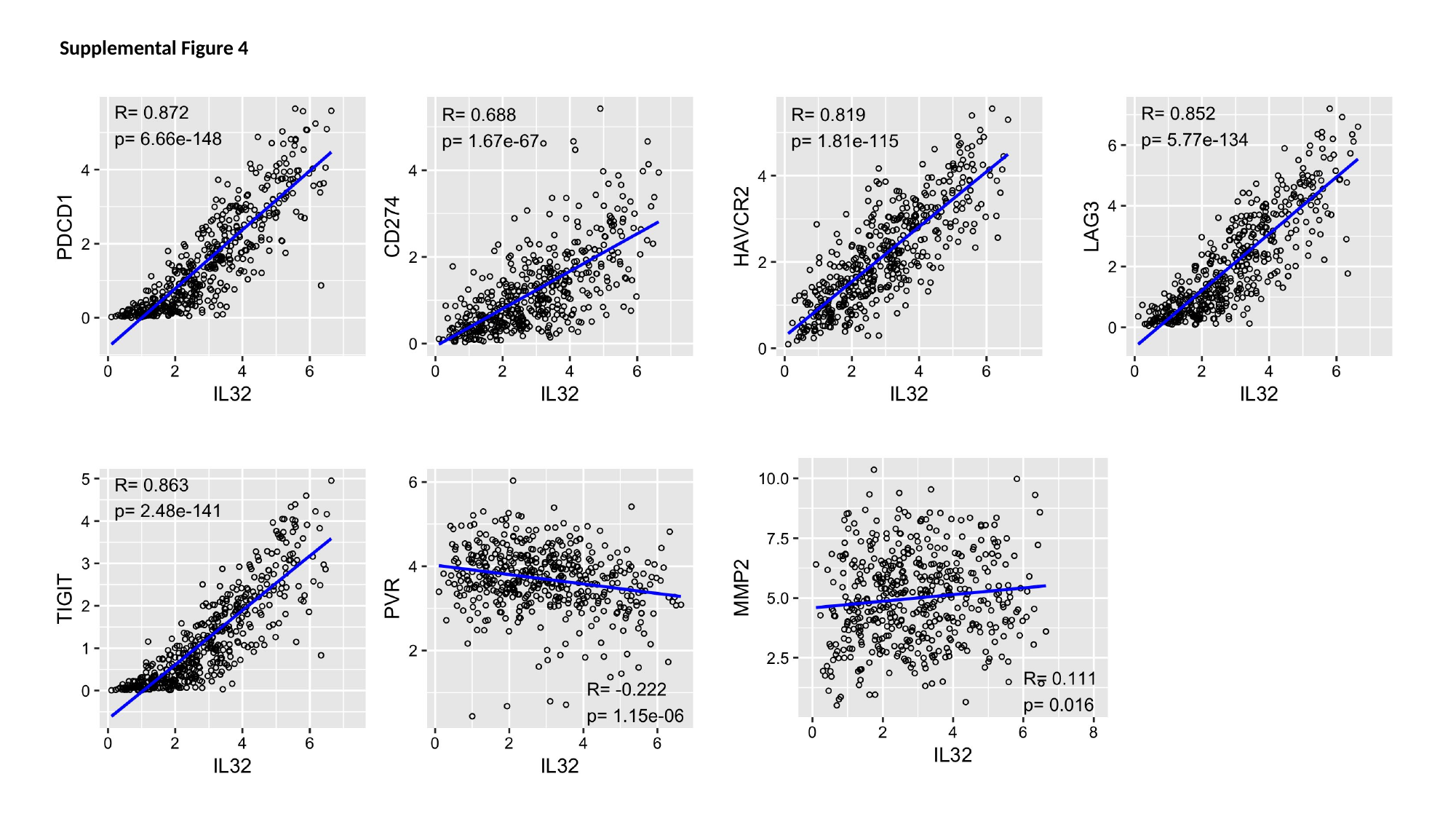

Supplemental Figure 4

Supplement: Supplementary file 4 — Additional file 4: Figure S4. IL32, checkpoint receptors/ligand expression, and markers of disease progression in the TCGA dataset. Scatterplot of log2 FPKM expression values between IL32 and select checkpoint receptors/ligands, as well as, select markers of disease progression (MMP2 and PVR). [file 12967_2019_1862_MOESM4_ESM.pptx]

## Slide 1
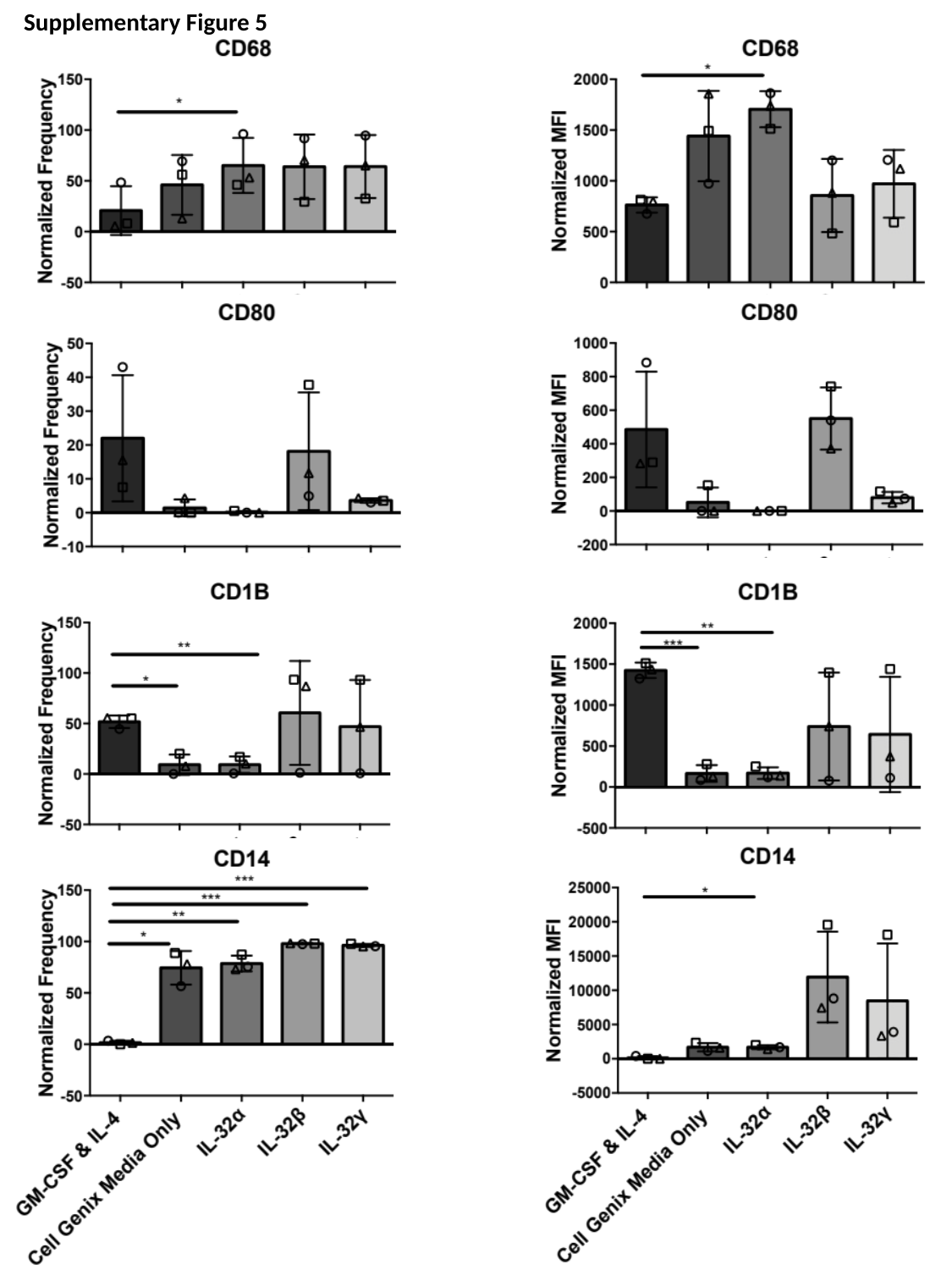

Supplementary Figure 5

Supplement: Supplementary file 5 — Additional file 5: Figure S5. Expression of phenotypic markers on human monocytes after exposure to stimuli. CD14 + cells, from PBMC isolation, were cultured in the presence of GM-CSF + IL-4, Cell Genix Media, recIL32α, recIL32β, or recIL32γ for 5 days. The different treatments are displayed by using a gray scale. On day 5, the phenotype of the cells was assayed using flow cytometry analysis for various surface markers. GM-CSF + IL-4 and Cell Genix Media alone were as a positive and negative control, respectively. Cells were then assessed for surface expression of CD68, CD80, CD14, CD1B by flow cytometry. Frequency of positive cells is shown in the left panels, and mean fluorescence intensity is shown in the right panels. Statistical analysis was done using a one-way repeated measures ANOVA, using the GM-CSF + IL-4 treatment as a control. Each shape (circle, square, triangle) represents a different healthy donor. *P ≤ 0.05, **P ≤ 0.01, ***P ≤ 0.001. [file 12967_2019_1862_MOESM5_ESM.pptx]
